# Combined transcriptomics and metabolomics analysis reveals the molecular responses of heat tolerance during germination stage in sesame

**DOI:** 10.3389/fpls.2026.1927626

**Published:** 2026-09-09

**Authors:** Pan Zeng, Yanxin Deng, Xiaowen Yan, Zhiqi Wang, Junchao Liang, Jian Sun

**Affiliations:** Jiangxi Province Key Laboratory of Oilcrops Genetic Improvement, Nanchang Branch of National Center of Oilcrops Improvement, Crops Institute of Jiangxi Academy of Agricultural Sciences, Nanchang, China

**Keywords:** sesame, heat stress, germination stage, metabolomic, transcriptomic, ERF, bHLH

## Abstract

Sesame is an important oilseed crop, but its germination stage growth is increasingly limited by heat stress under climate warming. However, the molecular responses underlying heat tolerance in sesame germination stage remain unclear. In this study, a heat-tolerant genotype (G14) and a heat-sensitive genotype (G9) were compared under different durations of high-temperature treatment using phenotypic, metabolomic, and transcriptomic analyses. The results showed that G14 maintained better growth than G9 under both normal and heat stress conditions, with a more pronounced advantage under high temperature. G14 also accumulated lower levels of hydrogen peroxide (H_2_O_2_) under heat stress, suggesting reduced oxidative damage and enhanced heat tolerance. Metabolomic analysis showed that differentially accumulated metabolites (DAMs) in G14 were mainly enriched in lipid metabolism, antioxidant systems, and secondary metabolism, which were activated at early stages and maintained throughout heat stress. In contrast, DAMs in G9 were primarily associated with hormone signaling, carbon metabolism, energy metabolism, and vitamin metabolism. Transcriptomic analysis further showed that heat stress induced extensive changes in gene expression. KEGG enrichment analysis suggested that differentially expressed genes (DEGs) in G14 were mainly involved in maintaining key physiological processes, including photosynthesis, protein processing, and lipid metabolism, whereas those in G9 were predominantly associated with defense and signaling pathways. Integrated metabolomic and transcriptomic analyses supported these patterns and indicated a close coordination between gene expression and metabolic changes. Transcription factor analysis identified members of the *ERF* and *bHLH* families as candidate transcription factors associated with the heat stress response in sesame. These findings provide new insights into the molecular responses underlying heat tolerance during sesame germination.

## 1 Introduction

Global warming has intensified in recent decades, making heat stress a major threat to crop productivity (Kan et al., 2023; Ren et al., 2026). It is estimated that, at the global scale, a 1 °C increase in temperature would reduce the yields of maize, wheat, soybean, and rice by 7.5 ± 5.3%, 6.0 ± 3.3%, 6.8 ± 5.9%, and 1.2 ± 5.2%, respectively (Hu et al., 2024). At the same time, the global population is projected to reach 10 billion by 2050, which will require a 60% increase in agricultural production to meet food demand (Ma et al., 2025). Therefore, understanding how plants respond to heat stress is essential for ensuring future food security.

High temperature disrupts cellular homeostasis at multiple levels. At the physiological level, heat stress damages photosystem II, reduces pigment content, and alters chloroplast structure, thereby limiting photosynthesis (Li et al., 2018). It also weakens membrane stability, causes protein misfolding, and leads to excessive accumulation of reactive oxygen species (ROS), which results in oxidative damage and even cell death under severe conditions (Hasanuzzaman et al., 2013; Correia et al., 2022). To cope with these effects, plants have developed complex signaling networks, including calcium signaling, phospholipid pathways, and mitogen-activated protein kinase (MAPK) cascades. These networks regulate the expression of stress-related genes and reshape metabolic pathways (Haider et al., 2021; Ding and Yang, 2022). Together, these changes determine the ability of plants to survive and grow under heat stress (Bhardwaj et al., 2015). Through both natural selection and breeding, plants have formed diverse regulatory systems to adapt to high temperatures (Guo et al., 2006; Ohama et al., 2017). In recent years, many studies have focused on these systems to improve crop thermotolerance (Kushwah et al., 2021; Xu et al., 2021).

Sesame (*Sesamum indicum* L.) is one of the oldest and most important oilseed crops in the world (Sharma et al., 2021). It is characterized by a high seed oil content, which can reach up to 63% in some varieties and is comparable to or higher than that of several major oil crops, including peanut (45–56%), sunflower (~45%), rapeseed (~40%), and soybean (~20%) (Rauf et al., 2024; Teklu et al., 2021). Sesame has a short growth cycle (70–90 days), high seed production (about 6,000 seeds per plant), and a relatively small genome (354 Mb). These features make it a useful model for studying stress responses and agronomic traits in plants (Zhang et al., 2019). Although sesame is mainly grown in tropical and subtropical regions in summer, and shows relatively strong tolerance to drought and heat, exposure to temperatures above 42°C can still severely limit its growth and reduce yield (Nimmakayala et al., 2011; Karimzadeh Soureshjani et al., 2019).

Recent studies have shown that heat-tolerant sesame varieties improve thermotolerance mainly by regulating hormone signaling pathways and heat shock protein (HSP) genes (Su et al., 2022). These varieties usually show higher antioxidant capacity, higher auxin levels, and lower abscisic acid (ABA) levels under heat stress (Su et al., 2024). However, the molecular basis underlying heat tolerance in sesame during the germination stage remains largely unclear. Based on existing research and our preliminary experiments, 44 °C was used as heat stress treatment (Su et al., 2022, 2024). Then, we identified a heat-tolerant genotype (G14) and a heat-sensitive genotype (G9). In the present study, these two genotypes were exposed to high temperature (44 °C), and their metabolic and transcriptional changes in shoots and roots during germination were investigated using metabolomic and transcriptomic approaches. This study provides new insights into the molecular responses associated with heat tolerance in sesame during the germination stage.

## 2 Materials and methods

### 2.1 Plant materials and treatment

A heat-tolerant genotype (G14) and a heat-sensitive genotype (G9), provided by Crops Institute of Jiangxi Academy of Agricultural Sciences, were used in this study. Seeds were surface-sterilized with 10% sodium hypochlorite, rinsed thoroughly, and soaked in water. They were then germinated in an incubator for 6 days, the conditions were set as follows: 30°C and dark during the day and night, 70% relative humidity.

For phenotypic analysis, seeds were germinated at 44°C for 7 days. Phenotypes were then recorded and photographed. Each experiment was repeated three times, with at least 10 biological replicates per treatment.

For transcriptomic and metabolomic analyses, 7-day-old seedlings grown under normal conditions were exposed to 44°C for 0 h, 12 h, and 24 h. The 12 h and 24 h time points were selected to capture early and later transcriptional and metabolic responses to heat stress, respectively, while avoiding excessively prolonged heat exposure that could compromise sample quality. A total of 12 groups were sampled: G14 shoots (G14-S0, G14-S12, G14-S24), G14 roots (G14-R0, G14-R12, G14-R24), G9 shoots (G9-S0, G9-S12, G9-S24), and G9 roots (G9-R0, G9-R12, G9-R24). All samples were rapidly frozen in liquid nitrogen and stored at −80°C for further analysis.

### 2.2 Determination of H_2_O_2_ content

The content of H_2_O_2_ were determined using commercial assay kits (Norminkoda, cat. no.NMW0106).

### 2.3 Transcriptome analysis

Total RNA was extracted following standard protocols used for RNA isolation. RNA quality was assessed using the RNA Nano 6000 Assay Kit on the Bioanalyzer 2100 system (Agilent, USA). For each sample, 1 μg of RNA was used for library construction and sequencing on the Illumina platform by Novogene (Beijing, China).

Raw data in FASTQ format were processed using FASTP to remove adapter sequences, reads with poly-N, and low-quality reads. Clean reads were then aligned to the sesame reference genome (ncbi_sesamum_indicum_gcf_000512975_1_s_indicum_v1-0) using HISAT2 (v2.2.1). Quality metrics, including Q20, Q30, and GC content, were calculated.

Gene expression levels were quantified as fragments per kilobase of transcript per million mapped reads (FPKM) using StringTie (v2.2.3) (Pertea et al., 2015). Differentially expressed genes (DEGs) were identified using DESeq2. *P*-values were adjusted using the Benjamini–Hochberg (BH) method to control the false discovery rate (FDR). Genes with |log2(fold change)| > 1 and adjusted *p*-value < 0.05 were defined as DEGs. KEGG pathway enrichment analysis was performed using the clusterProfiler package in R, and pathways with *p*-adjust < 0.05 were considered significantly enriched.

### 2.4 Metabolomic analysis

Each sample (100 mg) was ground in liquid nitrogen and extracted with 80% methanol. After vortexing and centrifugation, the supernatant was diluted to 53% methanol with LC-MS grade water. The mixture was centrifuged again at 15,000 × g at 4°C for 20 min. The final supernatant was used for LC-MS/MS analysis (Want et al., 2010).

Metabolite profiling was performed using a Vanquish UHPLC system coupled with an Orbitrap Q Exactive HF mass spectrometer (Thermo Fisher, Germany) at Novogene. Samples were separated on a Hypersil Gold column (100 × 2.1 mm, 1.9 μm) with a flow rate of 0.2 mL/min. The mobile phases were 0.1% formic acid in water (A) and methanol (B). The gradient program was as follows: 2% B for 1.5 min, 2–85% B over 3 min, 85–100% B over 10 min, 100–2% B over 10.1 min, and 2% B until 12 min.

The mass spectrometer was operated in both positive and negative ion modes. The spray voltage was set to 3.5 kV, capillary temperature to 320°C, sheath gas to 35 psi, auxiliary gas to 10 L/min, S-lens RF level to 60, and auxiliary gas heater temperature to 350 °C.

Metabolites were annotated using the KEGG (https://www.genome.jp/kegg/pathway.html), HMDB (https://hmdb.ca/metabolites), and LIPID MAPS (http://www.lipidmaps.org/) databases. Data were normalized and analyzed by principal component analysis (PCA) and partial least squares-discriminant analysis (PLS-DA) using metaX software (Wen et al., 2017). A t-test was used for statistical analysis. Metabolites with VIP > 1, *p*-value < 0.05, and fold change ≥ 2 or ≤ 0.5 were defined as differential metabolites. The functions of these metabolites and metabolic pathways were studied using the KEGG database. The metabolic pathways enrichment of differential metabolites was performed, when ratio were satisfied by x/n > y/N (n the total number of significantly altered metabolites, y the total number of metabolites assigned to that pathway in the reference database and N the total number of background metabolites used for enrichment analysis), metabolic pathway were considered as enrichment, when P-value of metabolic pathway < 0.05, metabolic pathway were considered as statistically significant enrichment.

### 2.5 Quantitative real-time PCR (RT-qPCR) analysis

Total RNA was reverse transcribed into first-strand cDNA using the RevertAid First Strand cDNA Synthesis Kit (Thermo Scientific, Waltham, MA, USA). RT-qPCR was performed using SYBR Green Real-time PCR Master Mix (Toyobo, Osaka, Japan) on a CFX96 Touch Real-Time PCR Detection System (Bio-Rad, Hercules, CA, USA). The thermal cycling conditions were as follows: an initial denaturation at 95°C for 30 s, followed by 40 cycles of 95°C for 10 s, 60°C for 30 s, and 72°C for 30 s. Relative gene expression levels were calculated using the 2^−ΔΔCt^ method with CFX Manager software (Bio-Rad, Hercules, CA, USA) (Livak and Schmittgen, 2001). Three biological replicates were analyzed for each sample, with three technical replicates for each biological replicate. Gene-specific primer sequences and detailed information are provided in **Supplementary Table 4**.

### 2.6 Integrative metabolomic-transcriptomic analysis

DEGs and DAMs from the same treatment comparisons were independently mapped to KEGG pathways. Pathways containing both DEGs and DAMs were identified as shared pathways and used for integrated transcriptomic and metabolomic analysis. For the integrated KEGG pathway analysis, pathways with a *p*-value < 0.05 were considered significantly enriched, based on the statistical criteria of the original analysis. No multiple-testing correction was applied to the integrated KEGG pathway analysis. The shared pathways were further compared across genotypes, tissues, and heat-stress durations to identify biological processes showing coordinated changes at the transcript and metabolite levels. Enrichment analysis plots were generated using R.

### 2.7 Transcription factor analysis

Heatmaps generated with TBtools-II were used to visualize differentially expressed transcription factors (Chen et al., 2023).

### 2.8 Statistical analysis

Statistical analyses were conducted using GraphPad Prism software (v10.6.0). Student’s *t*-test was used for comparisons between two groups based on independent biological replicates. Data are presented as means ± standard deviation (SD). A *p*-value < 0.05 was considered statistically significant (*p < 0.05; **p < 0.01; ns, not significant).

## 3 Results

### 3.1 Effects of different temperatures on sesame seedlings

Normal germination and high-temperature treatments were applied to G14 and G9. At 30°C, both shoot length and root length were greater in G14 than in G9 (**Figure 1A**). Specifically, the shoot length and root length of G14 were increased by 15.71% and 14.45%, respectively, compared with those of G9 (**Figures 1B, C**). At 44°C, growth was inhibited in both genotypes. However, G14 still maintained a clear growth advantage over G9 (**Figure 1A**). Compared with G14, G9 exhibited reductions of 38.07% and 67.65% in shoot length and root length, respectively, under heat stress (**Figures 1B, C**). The H_2_O_2_ contents in shoots and roots were measured separately to assess oxidative stress. In shoots, G9 accumulated significantly higher H_2_O_2_ levels than G14 under control conditions and after 12 h and 24 h of heat stress, with increases of 49.16%, 50.82%, and 21.12%, respectively. In roots, the H_2_O_2_ content of G9 was 21.6% higher than that of G14 under control conditions, whereas no significant differences were detected between the two genotypes after 12 h or 24 h of heat treatment (**Figures 1D, E**).

**Figure 1:**
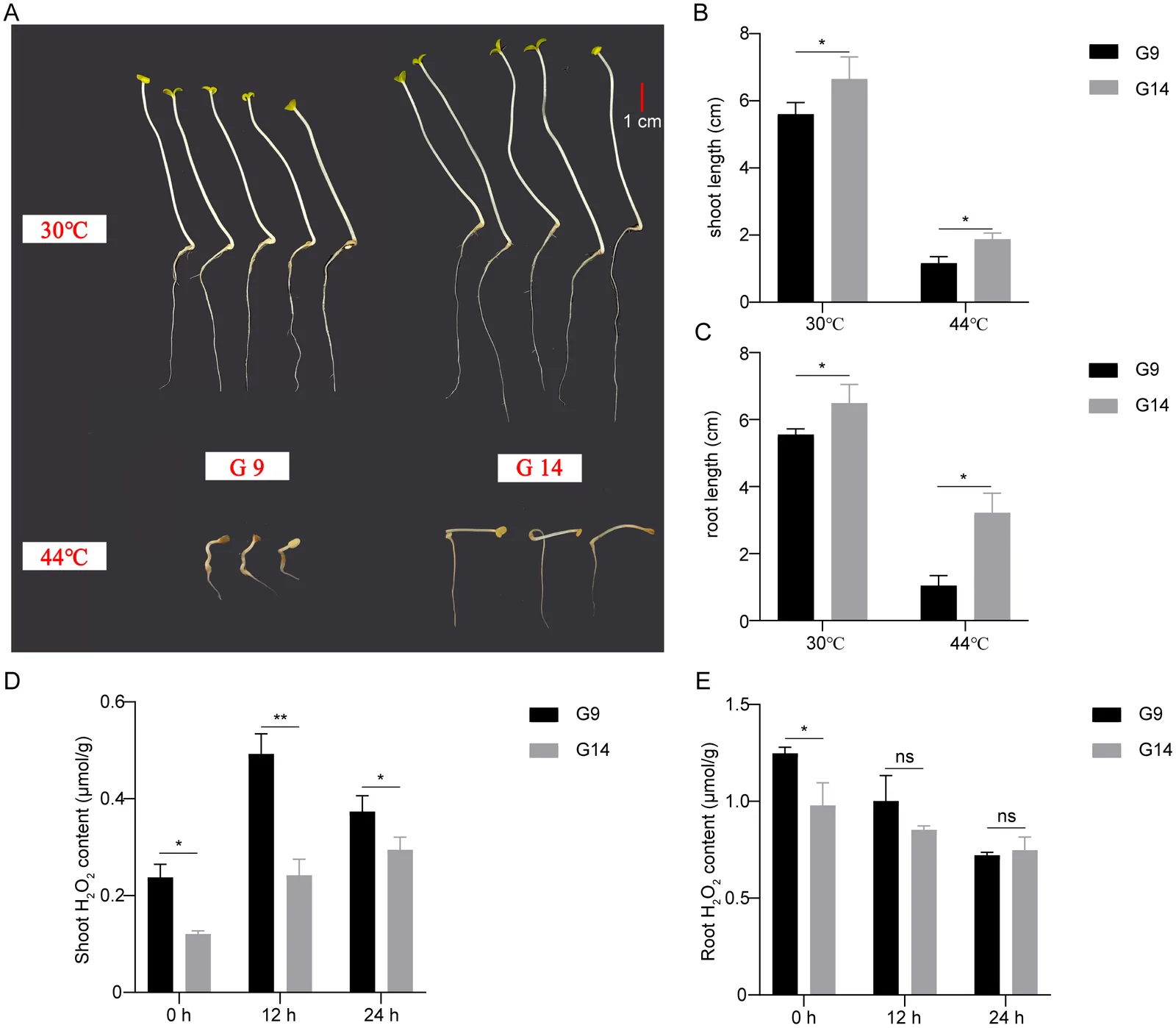
Phenotypes of G14 and G9 at 30°C and 44°C. **(A)** Growth phenotypes of G14 and G9 at 30°C and 44°C. G9 showed weaker growth than G14 under both conditions. Scale bar=1 cm. **(B, C)** Statistical analysis of shoot length **(B)** and root length **(C)**. Significant differences were analyzed by Student’s *t*-test (mean ± SD; *p < 0.05). **(D, E)** Statistical analysis of H_2_O_2_ content in shoot **(D)** and root **(E)**. Significant differences were analyzed by Student’s *t*-test (mean ± SD; *p < 0.05; **p < 0.01; ns, not significant).

Taken together, G14 exhibited better growth performance than G9 under both normal and high-temperature conditions, and this difference became more pronounced under heat stress. The lower H_2_O_2_ accumulation in G14 shoots under heat stress indicates a lower level of oxidative stress than in G9. However, H_2_O_2_ measurements alone provide evidence only for differences in oxidative stress status and do not fully represent heat tolerance. The differences in growth performance, together with the H_2_O_2_ measurements, provide physiological evidence for the differential responses of G14 and G9 to heat stress and support further investigation of the molecular basis of their heat responses.

### 3.2 Quantitative analysis of differential metabolites

To examine the metabolic response of sesame to heat stress, after germinating for 6 days under control conditions (30°C), the sesame seedlings were transferred to a growth chamber for heat stress exposure at 44 °C, and we untargeted metabolomic analysis was performed on G14 and G9 at 0 h, 12 h, and 24 h. Both genotypes showed clear metabolic changes at different time points, but their response patterns differed.

In positive ion mode, G14 shoots contained 206 (63 up-regulated and 143 down-regulated) and 297 (68 up-regulated and 229 down-regulated) differential metabolites at 12 h and 24 h, respectively (**Figure 2A**). In roots, 211 (96 up-regulated and 115 down-regulated) and 294 (92 up-regulated and 202 down-regulated) metabolites were detected (**Figure 2A**). In contrast, G9 showed higher numbers. In shoots, 308 (85 up-regulated and 223 down-regulated) and 333 (52 up-regulated and 281 down-regulated) metabolites were identified at 12 h and 24 h, respectively (**Figure 2B**). In roots, the numbers were 281 (107 up-regulated and 174 down-regulated) and 286 (93 up-regulated and 193 down-regulated) (**Figure 2B**). Overall, G9 showed more differential metabolites at each time point, with most being down-regulated. These results indicate that heat stress induced broader metabolic alterations in G9 than in G14.

**Figure 2:**
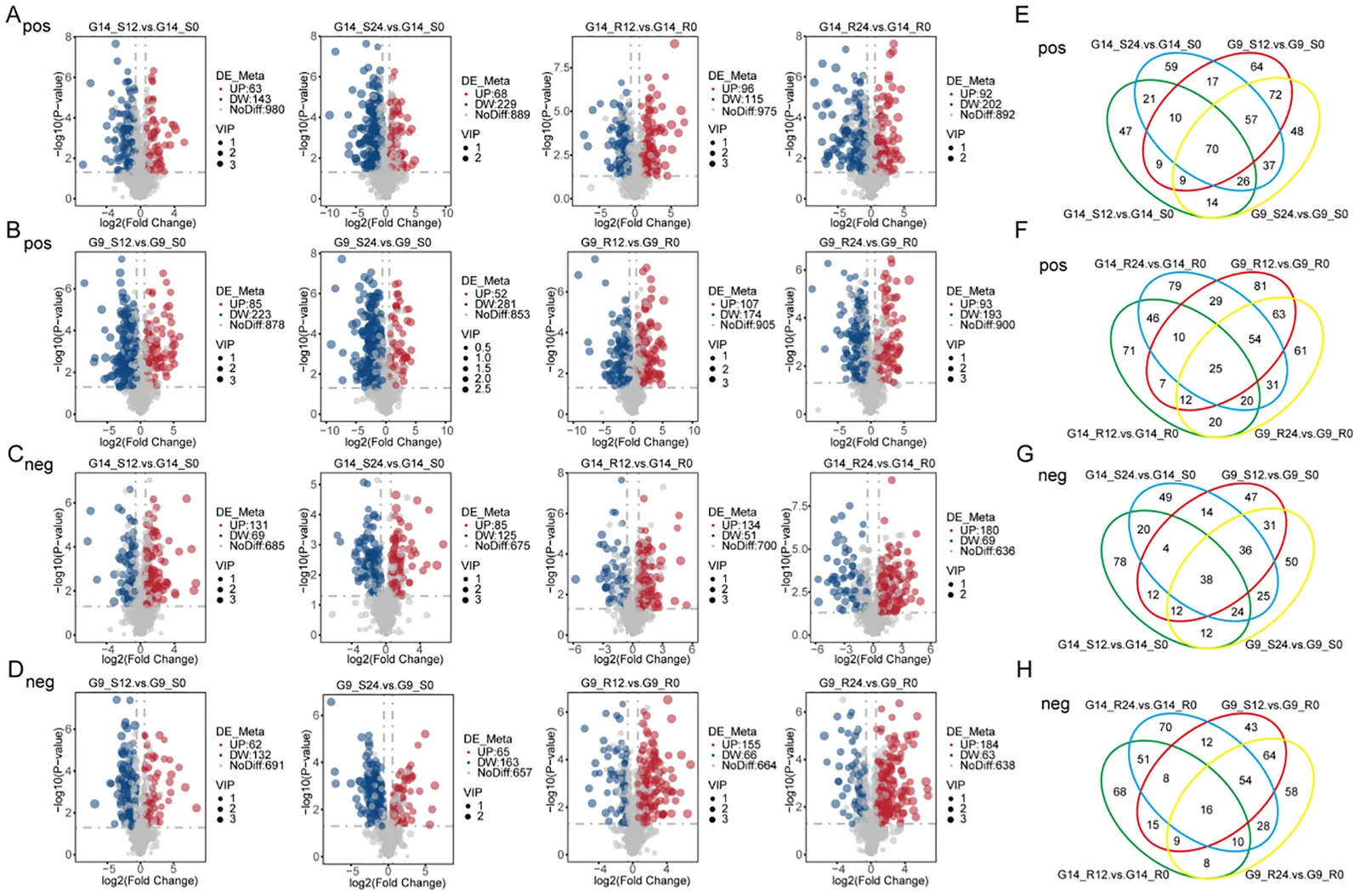
Differential metabolite analysis in G14 and G9 under heat stress. **(A, B)** Volcano plots of differential metabolites in shoots and roots of G14 **(A)** and G9 **(B)** at 12 h and 24 h under positive ion mode. **(C, D)** Volcano plots of differential metabolites in shoots and roots of G14 **(C)** and G9 **(D)** at 12 h and 24 h under negative ion mode. **(E, F)** Venn diagrams of differential metabolites in shoots **(E)** and roots **(F)** of G14 and G9 under positive ion mode. G-H. Venn diagrams of differential metabolites in shoots **(G)** and roots **(H)** of G14 and G9 under negative ion mode.

In negative ion mode, G14 shoots contained 200 (69 up-regulated and 131 down-regulated) and 210 (85 up-regulated and 125 down-regulated) metabolites at 12 h and 24 h (**Figure 2C**). Roots showed 185 (134 up-regulated and 51 down-regulated) and 249 (180 up-regulated and 69 down-regulated) metabolites (**Figure 2C**). G9 shoots contained 194 (62 up-regulated and 132 down-regulated) and 228 (65 up-regulated and 163 down-regulated) metabolites, while roots contained 221 (155 up-regulated and 66 down-regulated) and 247 (184 up-regulated and 63 down-regulated) (**Figure 2D**). Notably, in this mode, both genotypes showed mainly up-regulated metabolites in roots. This indicates that roots may respond to heat stress by activating specific metabolic pathways.

Comparison of overlapping metabolites showed that, in positive ion mode, G14 shoots and roots shared 127 and 101 metabolites between 12 h and 24 h, respectively (**Figures 2E, F**). In G9, the numbers were higher, with 208 in shoots and 154 in roots (**Figures 2E, F**). Between genotypes, 70 common metabolites were detected in shoots and 25 in roots (**Figures 2E, F**). In negative ion mode, G14 shared 86 and 85 metabolites in shoots and roots, while G9 shared 117 and 143. Between genotypes, 38 common metabolites were found in shoots and 16 in roots (**Figures 2G, H**).

Taken together, G9 showed more shared metabolites across time points, indicating a more sustained metabolic response under heat stress. The limited overlap between G14 and G9 suggests that the two genotypes employed distinct metabolic responses to cope with heat stress. Overall, heat stress induced extensive metabolic changes during sesame germination. Compared with G9, G14 showed fewer metabolic alterations and a relatively stable metabolic profile, which may be related to its superior growth performance under heat stress.

### 3.3 KEGG analysis of differential metabolites

In positive ion mode, pathway enrichment analysis revealed that both G14 and G9 shoots shared several early responses to heat stress at 12 h, mainly involving. In particular, Linoleic acid metabolism, alpha-Linolenic acid metabolism, and Glutathione metabolism were consistently enriched, suggesting that both genotypes activated lipid-related metabolic processes and antioxidant mechanisms during the early phase of heat stress. In addition, pathways associated with carbon and nitrogen metabolism, including Arginine and proline metabolism and Fructose and mannose metabolism, were enriched, indicating adjustments in energy supply and osmotic regulation (**Figure 3A**).

**Figure 3:**
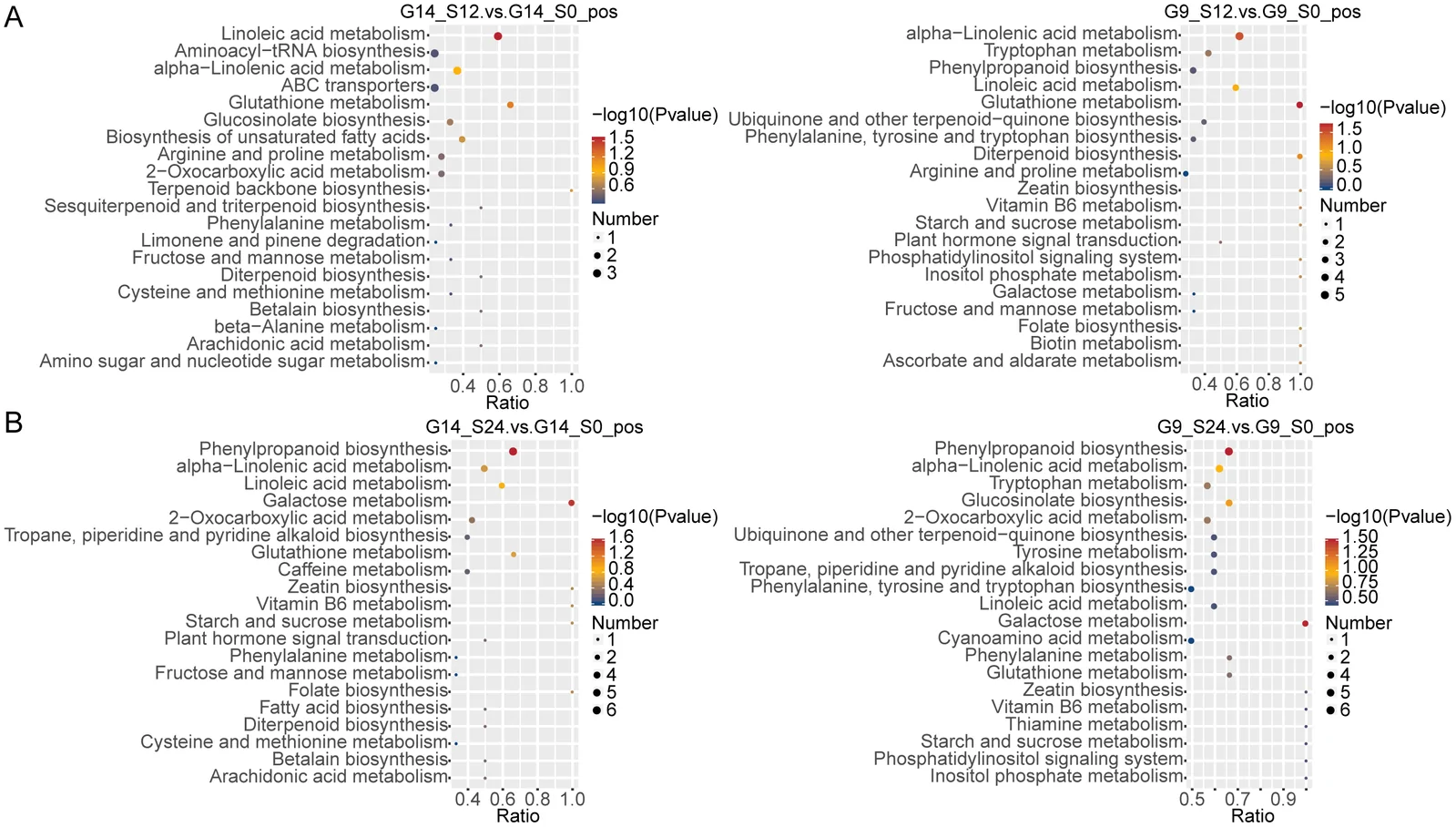
KEGG enrichment analysis of differential metabolites in G14 and G9 in positive ion mode. **(A, B)** KEGG enrichment bubble plots of differential metabolites in shoots of G14 and G9 after 12 h **(A)** and 24 h **(B)** of heat treatment.

Despite these common responses, the two genotypes exhibited distinct metabolic patterns. G14 showed stronger enrichment of related to lipid remodeling and secondary metabolism, including Terpenoid backbone biosynthesis, Biosynthesis of unsaturated fatty acids, and Arachidonic acid metabolism. These changes suggest that G14 showed stronger metabolic adjustment in lipid-related pathways and secondary metabolism. G14 also showed enrichment of Aminoacyl-tRNA biosynthesis and Cysteine and methionine metabolism, indicating active regulation of protein synthesis and sulfur-containing amino acid metabolism under heat stress. In contrast, G9 showed greater enrichment of pathways related to carbohydrate metabolism, hormone signaling, and vitamin metabolism, including Starch and sucrose metabolism, Plant hormone signal transduction, and Ascorbate and aldarate metabolism, suggesting a broader adjustment of metabolic processes in response to heat stress (**Figure 3A**).

At 24 h, both genotypes-maintained enrichment of Linoleic acid metabolism, alpha-Linolenic acid metabolism, and Glutathione metabolism in shoots, indicating sustained regulation of lipid-related metabolism and antioxidant capacity during prolonged heat exposure. Phenylpropanoid biosynthesis was also enriched, suggesting increased involvement of secondary metabolism and cell wall-related processes at later stages. G14 continued to exhibit enrichment of fatty acid-related pathways and Cysteine and methionine metabolism, whereas G9 showed stronger associations with Inositol phosphate metabolism, Phosphatidylinositol signaling, and amino acid metabolism. These results indicate that G14 maintained a relatively stable metabolic adjustment pattern, whereas G9 exhibited more diverse metabolic changes during heat stress (**Figure 3B**).

In roots, both genotypes showed early enrichment of pathways associated with secondary metabolism, hormone regulation, and amino acid metabolism. At 12 h, pathways involved in alkaloid biosynthesis, Glucosinolate biosynthesis, Flavonoid biosynthesis, and Plant hormone signal transduction were commonly enriched, suggesting that roots rapidly activated defense-related metabolic pathways. G14 showed additional enrichment of Sphingolipid metabolism and Linoleic acid metabolism, indicating stronger changes in lipid-related metabolic pathways. In contrast, G9 showed greater enrichment of primary metabolic pathways, including Starch and sucrose metabolism and Fatty acid biosynthesis, suggesting stronger metabolic adjustments to maintain basic cellular processes under heat stress (**Supplementary Figure 3A**). At 24 h, both genotypes continued to enrich pathways related to secondary metabolism and carbohydrate metabolism. G14 maintained enrichment of fatty acid-related pathways, including Fatty acid biosynthesis and Biosynthesis of unsaturated fatty acids, whereas G9 showed increased enrichment of antioxidant-related pathways, such as Glutathione metabolism, α-Linolenic acid metabolism, and Ascorbate and aldarate metabolism. This difference suggests that G14 may rely more on maintaining a relatively stable metabolic response, while G9 may require additional activation of protective pathways during prolonged heat exposure (**Supplementary Figure 3B**).

The enrichment patterns observed in negative ion mode were generally consistent with those in positive ion mode, although energy metabolism pathways were more prominent. In shoots, both genotypes showed enrichment of pathways related to nucleotide metabolism, amino acid biosynthesis, and carbohydrate metabolism at early stages. G14 showed stronger associations with carbon metabolism and glycolysis/gluconeogenesis, whereas G9 was more enriched in TCA cycle and pyruvate metabolism, indicating differences in energy metabolism regulation between the two genotypes. At later stages, G14 showed increased enrichment of flavonoid biosynthesis, glutathione metabolism, and secondary metabolism, while G9 remained associated with carbohydrate and energy metabolism pathways (**Supplementary Figures 1A, B**).

In roots, both genotypes exhibited enrichment of pathways related to amino acid metabolism, coenzyme metabolism, and secondary metabolism. G14 showed greater involvement of carbohydrate metabolism, nitrogen metabolism, and unsaturated fatty acid biosynthesis, whereas G9 showed stronger enrichment of hormone signaling, glutathione metabolism, and glycolysis/gluconeogenesis. At 24 h, G14 maintained enrichment of glutathione metabolism and fatty acid metabolism, while G9 showed increased enrichment of hormone signaling and secondary metabolism (**Supplementary Figures 1C, D**).

Overall, both genotypes shared conserved metabolic responses to heat stress, particularly involving lipid metabolism, antioxidant processes, carbon metabolism, and amino acid metabolism. However, their response strategies differed. G14 showed a relatively coordinated metabolic pattern characterized by sustained regulation of lipid metabolism, antioxidant-related pathways, and secondary metabolism. In contrast, G9 displayed broader metabolic alterations involving energy metabolism, hormone signaling, and carbohydrate metabolism. These differences suggest that heat tolerance in G14 may be associated with a more stable metabolic response, whereas G9 undergoes more extensive metabolic adjustments under heat stress.

### 3.4 Quantitative analysis of differential genes

To better understand the molecular response of sesame to heat stress, transcriptome sequencing was performed on samples collected at different treatment times. The sequencing data showed high quality and reliability. A total of 1,567,075,514 clean reads were obtained from 36 samples, with an average sequencing depth exceeding 20× per sample and approximately 6 GB of data generated for each (**Supplementary Table 1**). All samples exhibited Q30 values above 96.5%, GC contents above 44.5% and the alignment rates were more than 85%, indicating high sequencing accuracy and consistency (**Supplementary Table 1**). Principal component analysis (PCA) revealed distinct clustering patterns among different treatment groups (**Figure 4A**). The first two principal components explained 73.85% of the total variance, with PC1 and PC2 accounting for 45.41% and 28.44% of the variation, respectively. Samples from the same treatment group clustered closely together, whereas heat-treated samples were clearly separated from control samples, indicating that heat stress induced substantial changes in global gene expression profiles (**Figure 4A**). Together, these results demonstrate the reliability of the transcriptome data and provide a solid foundation for further analysis of heat-responsive genes in sesame.

**Figure 4:**
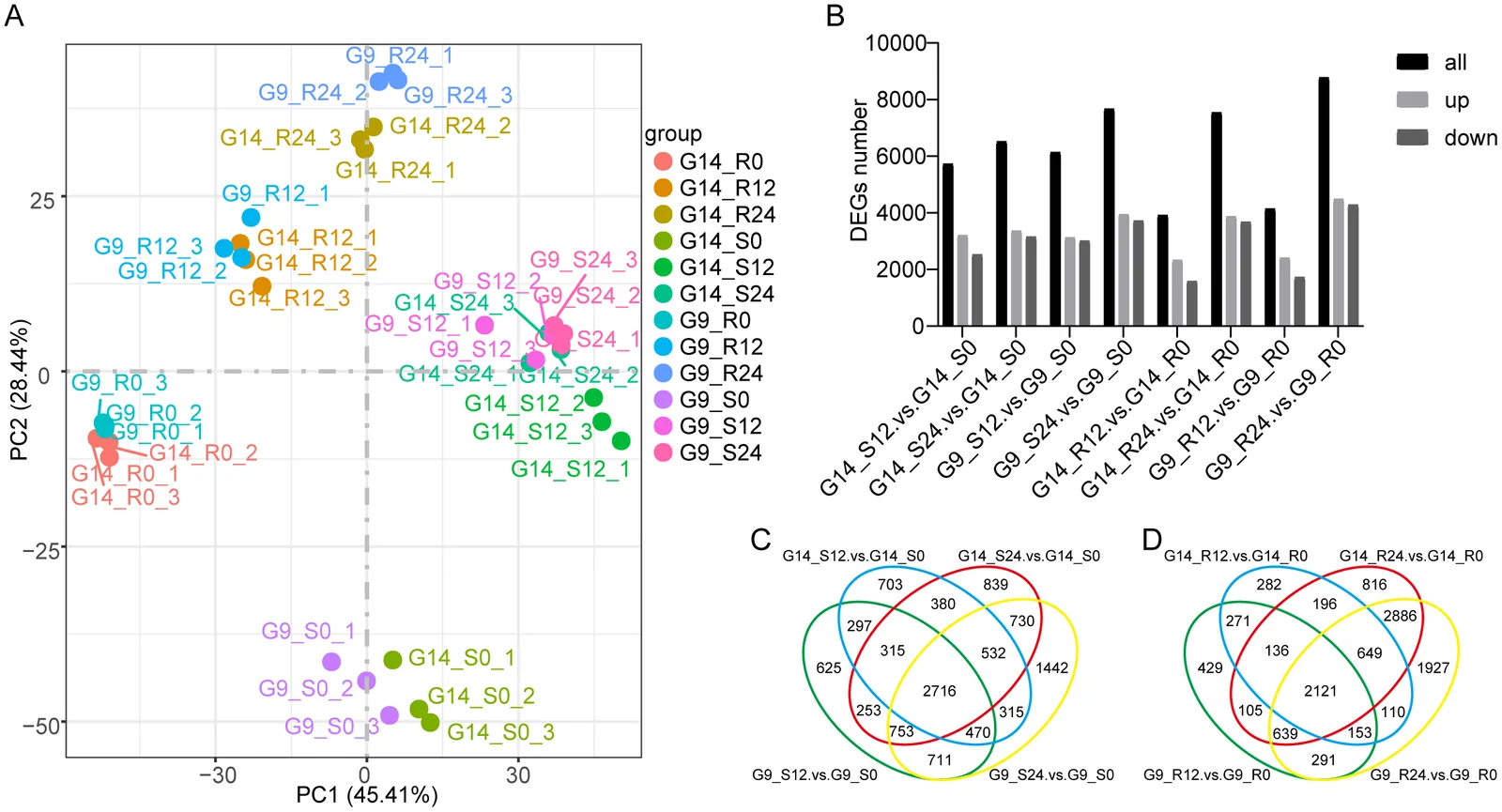
Transcriptome PCA analysis and differential gene analysis **(A, B)**. **(A)** PCA analysis of 36 samples. **(B)** Number of DEGs under different treatments. **(C, D)** Venn diagram showing shared and unique DEGs in shoots **(C)** and roots **(D)** of G14 and G9 under heat treatment for 12 h and 24 h.

Analysis of DEGs showed clear differences between the two genotypes. In shoots, G14 had 5,728 (3,200 up-regulated and 2,528 down-regulated) DEGs at 12 h and 6,518 (3,365 up-regulated and 3,153 down-regulated) at 24 h. In roots, the numbers were 3,918 (2,329 up-regulated and 1,589 down-regulated) at 12 h and 7,548 (3,869 up-regulated and 3,679 down-regulated) at 24 h. In comparison, G9 showed higher DEG numbers. In shoots, 6,140 (3,131 up-regulated and 3,009 down-regulated) DEGs were detected at 12 h and 7,669 (3,944 up-regulated and 3,725 down-regulated) at 24 h. In roots, 4,145 (2,416 up-regulated and 1,729 down-regulated) and 8,776 (4,490 up-regulated and 4,286 down-regulated) DEGs were identified at 12 h and 24 h, respectively (**Figure 4B**).

Overall, G9 showed more extensive transcriptional changes than G14 under heat stress, as indicated by the higher number of DEGs at both time points. These differences suggest that the two genotypes adopted distinct transcriptional response patterns under high-temperature conditions. In addition, 2,716 and 2,121 common DEGs were identified in shoots and roots, respectively (**Figures 4C, D**). These genes may form a core regulatory network involved in the heat stress response.

In summary, heat stress induced large-scale transcriptional reprogramming in sesame. The two genotypes differed in both response strength and regulatory pattern. These differences likely contribute to their contrasting heat tolerance.

### 3.5 KEGG analysis of differential genes

KEGG analysis showed clear differences in pathway enrichment between the two genotypes. At 12 h, both G14 and G9 shoots were significantly enriched in Photosynthesis - antenna proteins, Motor proteins, Protein processing in endoplasmic reticulum, and DNA replication. This indicates that both genotypes adjusted photosystem structure, protein folding, and cell division at the early stage of heat stress. In addition, G14 showed specific enrichment of Photosynthesis and Phenylpropanoid biosynthesis, suggesting that the heat-tolerant genotype preferentially maintained photosynthetic activity and activated secondary metabolic pathways involved in stress adaptation. In contrast, G9 was mainly enriched in Plant–pathogen interaction, Diterpenoid biosynthesis, Plant hormone signal transduction, and Carotenoid biosynthesis, indicating a broader activation of defense-related signaling and protective metabolic pathways in response to heat stress (**Figure 5A**).

**Figure 5:**
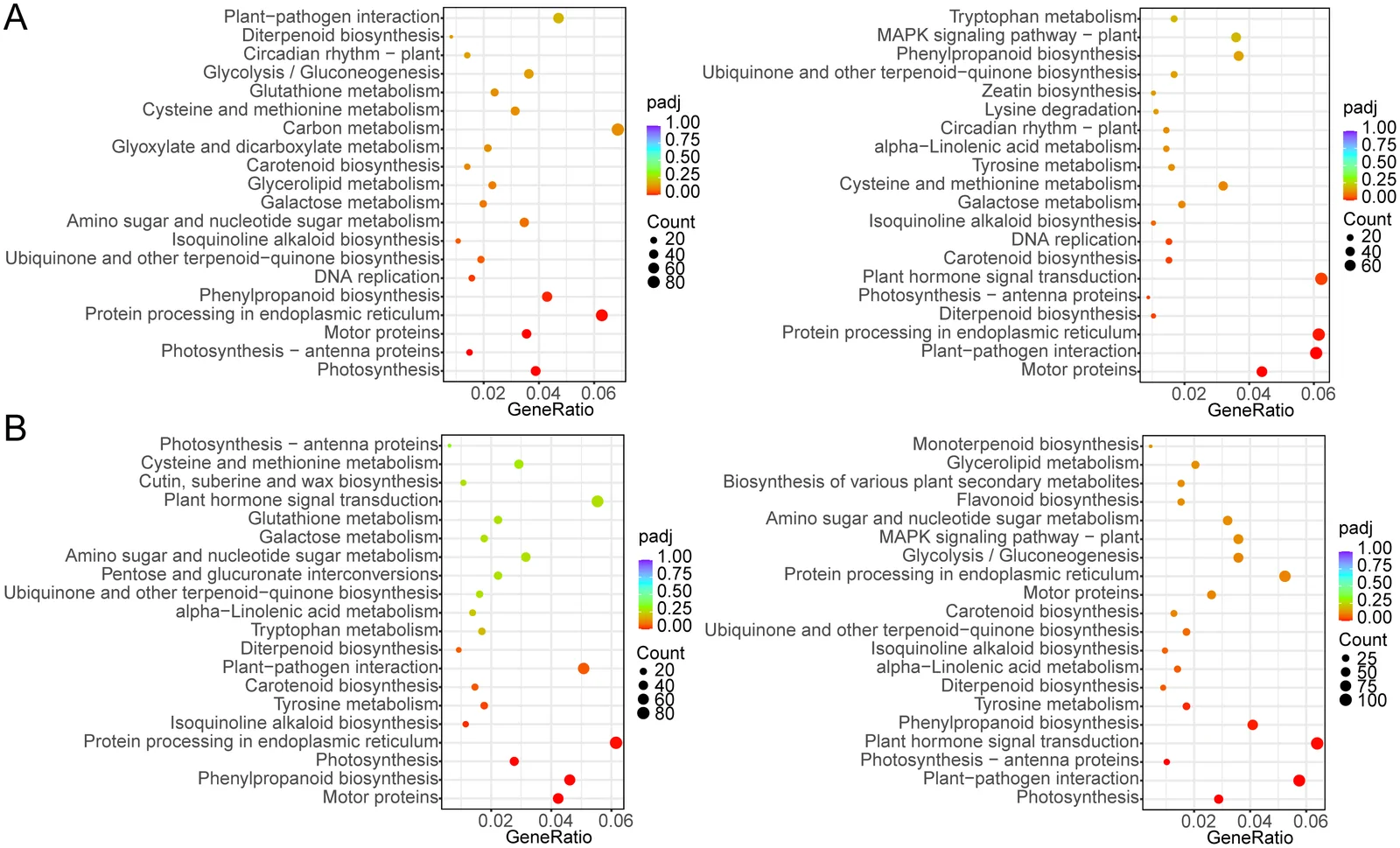
KEGG enrichment analysis of DEGs in G14 and G9. **(A, B)** KEGG enrichment bubble plots of DEGs in shoots of G14 and G9 after 12 h **(A)** and 24 h **(B)** of heat treatment.

At 24 h, both genotypes showed common enrichment in Phenylpropanoid biosynthesis, Photosynthesis, Tyrosine metabolism, and Plant–pathogen interaction. This suggests that secondary metabolism and defense responses became more active at later stages. G14 remained enriched in Motor proteins, Protein processing in endoplasmic reticulum, and Isoquinoline alkaloid biosynthesis, indicating sustained regulation of protein quality control and secondary metabolite production. G9, however, was mainly enriched in Photosynthesis - antenna proteins and Plant hormone signal transduction, suggesting continued adjustment of light-harvesting processes and hormone-mediated stress responses (**Figure 5B**).

In roots, both genotypes showed enrichment of pathways involved in Protein processing, secondary metabolism, transport, and physiological regulation at 12 h, including Protein processing in endoplasmic reticulum, Photosynthesis, Isoquinoline alkaloid biosynthesis, Tyrosine metabolism, ABC transporters, and Circadian rhythm - plant. These responses suggest that roots rapidly adjusted protein homeostasis, metabolite transport, and regulatory processes during the early stage of heat stress. G14 showed additional enrichment in Glycerolipid metabolism and Phenylpropanoid biosynthesis, indicating stronger changes in lipid-related metabolism and phenylpropanoid biosynthesis. In contrast, G9 showed stronger enrichment of pathways associated with carbohydrate metabolism, terpenoid biosynthesis, carotenoid biosynthesis, and hormone-related processes, suggesting a wider metabolic adjustment to heat stress (**Supplementary Figure 4A**).

At 24 h, both genotypes shared enrichment in Phenylpropanoid biosynthesis, Flavonoid biosynthesis, Plant–pathogen interaction, Ubiquinone and other terpenoid-quinone biosynthesis, Motor proteins, Zeatin biosynthesis, Tyrosine metabolism, and Glycolysis/Gluconeogenesis. These results indicate that secondary metabolism, defense, and energy metabolism dominated the later response in roots. G14 was further enriched in Photosynthesis, Fatty acid degradation, Sesquiterpenoid and triterpenoid biosynthesis, and ABC transporters, suggesting sustained regulation of photosynthetic processes, lipid metabolism, and metabolite transport. In contrast, G9 showed stronger enrichment of Carbon metabolism, Pyruvate metabolism, Isoquinoline alkaloid biosynthesis, and Galactose metabolism, indicating increased involvement of carbohydrate utilization and energy metabolism under prolonged heat stress (**Supplementary Figure 4B**).

Overall, both G14 and G9 activated conserved heat-responsive pathways involving photosynthesis, protein processing, secondary metabolism, and energy metabolism. However, their transcriptional responses showed distinct characteristics. G14 exhibited relatively consistent enrichment of pathways associated with photosynthetic regulation, protein homeostasis, lipid metabolism, and secondary metabolism, which may be associated with the maintenance of physiological functions under heat stress. In contrast, G9 showed broader involvement of hormone signaling, carbohydrate metabolism, and defense-related pathways, suggesting greater transcriptional adjustment to cope with heat stress. These differences indicate that the two genotypes employ distinct molecular strategies in response to high-temperature conditions. The enrichment of lipid-related pathways in G14 may be relevant to its heat response; however, whether these changes contribute directly to membrane stability or protection remains to be experimentally determined.

### 3.6 Integrated analysis of metabolome and transcriptome

The combined analysis of metabolomic and transcriptomic data revealed a close link between gene expression and metabolic changes under heat stress.

In positive ion mode, at 12 h, both G14 and G9 shoots were enriched in lipid metabolism, antioxidant defense, and primary metabolism, including Linoleic acid metabolism, Glutathione metabolism, alpha-Linolenic acid metabolism, Diterpenoid biosynthesis, Arginine and proline metabolism, 2-Oxocarboxylic acid metabolism, Fructose and mannose metabolism, Phenylalanine metabolism, Ubiquinone and other terpenoid-quinone biosynthesis, Phenylpropanoid biosynthesis, and amino acid biosynthesis pathways. These results indicate that early heat stress triggered coordinated adjustments in lipid-related metabolism, antioxidant responses, and amino acid metabolism. In particular, the enrichment of Linoleic acid metabolism and α-Linolenic acid metabolism suggests that lipid remodeling and lipid-derived signaling may be associated with the early response to high temperature, while Glutathione metabolism suggests an enhanced antioxidant response under heat stress. On this basis, G14 showed additional enrichment in lipid and secondary metabolism, including Terpenoid backbone biosynthesis, Biosynthesis of unsaturated fatty acids, Arachidonic acid metabolism, and Sesquiterpenoid and triterpenoid biosynthesis. These pathways are related to lipid metabolism and secondary metabolite biosynthesis, suggesting that G14 showed stronger metabolic responses in these pathways under heat stress. G14 also showed enrichment of Aminoacyl-tRNA biosynthesis and ABC transporters, indicating active regulation of protein synthesis and metabolite transport under heat stress. In contrast, G9 was more enriched in Ascorbate and aldarate metabolism, Starch and sucrose metabolism, Inositol phosphate metabolism, and Plant hormone signal transduction, suggesting broader adjustments in carbohydrate metabolism and stress signaling pathways (**Figure 6A**).

**Figure 6:**
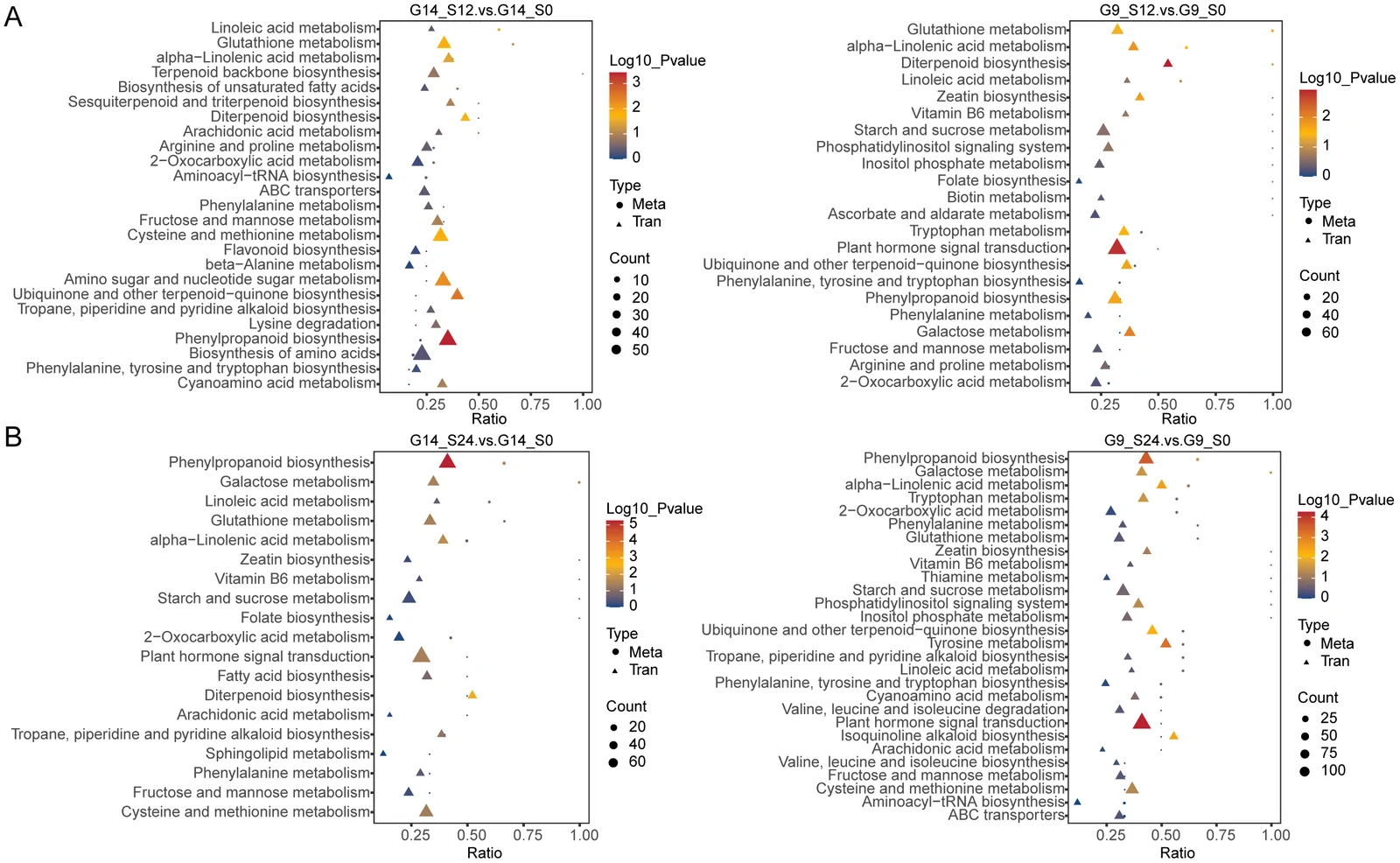
Integrated KEGG enrichment analysis of metabolome and transcriptome in positive ion mode. **(A, B)** Integrated KEGG enrichment bubble plots in shoots of G14 and G9 after 12 h **(A)** and 24 h **(B)**.

At 24 h, both genotypes showed common enrichment in Phenylpropanoid biosynthesis, Galactose metabolism, Linoleic acid metabolism, Glutathione metabolism, alpha-Linolenic acid metabolism, and Plant hormone signal transduction in shoots. The continued enrichment of Glutathione metabolism and lipid-related pathways indicates that antioxidant responses and lipid-related metabolic processes remained active during prolonged heat exposure. Phenylpropanoid metabolism may be associated with changes in secondary metabolism during heat stress. G14 was further enriched in Fatty acid biosynthesis, Diterpenoid biosynthesis, and Sphingolipid metabolism, suggesting sustained changes in lipid-related metabolism. In contrast, G9 was more associated with Inositol phosphate metabolism, Phosphatidylinositol signaling, and amino acid metabolism, reflecting increased involvement of signaling regulation and metabolic adjustment at later stages (**Figure 6B**).

In roots, at 12 h, both genotypes were enriched in pathways associated with secondary metabolism, hormone regulation, and metabolite transport, including Tropane, piperidine and pyridine alkaloid biosynthesis, Flavonoid biosynthesis, Folate biosynthesis, branched-chain amino acid degradation, Plant hormone signal transduction, and ABC transporters. These results indicate that roots rapidly activated secondary metabolic and signaling responses after heat exposure. G14 further enriched Linoleic acid metabolism, Sphingolipid metabolism, and Phenylpropanoid biosynthesis, suggesting stronger changes in lipid-related metabolism and secondary metabolism. In contrast, G9 showed stronger enrichment of Starch and sucrose metabolism, Fatty acid biosynthesis, and Glutathione metabolism, indicating increased metabolic adjustments to cope with heat-induced stress (**Supplementary Figure 5A**). At 24 h, both genotypes shared enrichment in Tropane, piperidine and pyridine alkaloid biosynthesis, Terpenoid backbone biosynthesis, Amino sugar and nucleotide sugar metabolism, and Phenylalanine metabolism in roots. G14 further enriched Fatty acid biosynthesis and Biosynthesis of unsaturated fatty acids, suggesting continued changes in lipid metabolism under prolonged heat stress. In contrast, G9 showed additional enrichment of antioxidant pathways such as Glutathione metabolism, alpha-Linolenic acid metabolism, and Ascorbate and aldarate metabolism, suggesting increased activation of antioxidant-related responses at later stages (**Supplementary Figure 5B**).

In negative ion mode, the overall pattern was similar, but energy metabolism pathways were more prominent. At 12 h, both genotypes showed enrichment in Purine metabolism, Biosynthesis of amino acids, Starch and sucrose metabolism, and Carbon metabolism in shoots, indicating adjustments in energy production and metabolic supply during heat stress. G14 further enriched Glycolysis/Gluconeogenesis, Carbon fixation, and Glutathione metabolism, suggesting coordinated regulation of carbon utilization and antioxidant-related responses. In contrast, G9 was more associated with the Citrate cycle, Pyruvate metabolism, and amino acid metabolism, indicating greater involvement of energy metabolism pathways (**Supplementary Figure 2A**). At 24 h, both genotypes were enriched in Amino sugar and nucleotide sugar metabolism, Linoleic acid metabolism, Pantothenate and CoA biosynthesis, Glutathione metabolism, and Plant hormone signal transduction in shoots. G14 further enriched Flavonoid biosynthesis and Arginine and proline metabolism, which are related to secondary metabolism and osmotic adjustment. Meanwhile, G9 remained mainly enriched in Carbon metabolism, the Citrate cycle, and Glycolysis/Gluconeogenesis, suggesting continued adjustment of energy metabolism under prolonged heat stress (**Supplementary Figure 2B**).

In roots, both genotypes showed enrichment of Glutathione metabolism, Arginine biosynthesis, Pantothenate and CoA biosynthesis, and alkaloid biosynthesis at 12 h. G14 further enriched Starch and sucrose metabolism, Nitrogen metabolism, and Oxidative phosphorylation, indicating enhanced regulation of carbon and energy metabolism. In contrast, G9 was more associated with Glycolysis/Gluconeogenesis, alpha-Linolenic acid metabolism, and secondary metabolism (**Supplementary Figure 2C**). At 24 h, both genotypes shared enrichment in Biosynthesis of amino acids, Fatty acid degradation, Oxidative phosphorylation, and Glutathione metabolism in roots. G14 further enriched Glycolysis/Gluconeogenesis, Fatty acid metabolism, and Ascorbate and aldarate metabolism, whereas G9 was mainly enriched in Plant hormone signal transduction and amino acid metabolism (**Supplementary Figure 2D**).

Overall, the integrated analysis demonstrated that both genotypes activated conserved metabolic responses involving lipid metabolism, antioxidant defense, carbon metabolism, and amino acid metabolism under heat stress. Among these pathways, Glutathione metabolism was frequently enriched across different tissues, treatment durations, and analytical modes, suggesting that glutathione-related metabolism may be associated with the regulation of redox status during heat exposure. In addition, the continuous regulation of lipid metabolism and secondary metabolic pathways suggests their potential relevance to the heat stress response, although their direct roles in membrane protection have not been experimentally validated. However, G14 exhibited a relatively coordinated response characterized by sustained regulation of lipid metabolism, antioxidant-related pathways, and secondary metabolism, whereas G9 showed broader metabolic adjustments involving hormone signaling and energy metabolism. These distinct metabolic response patterns may be associated with differences in heat tolerance between the two genotypes.

### 3.7 Changes in differentially expressed transcription factors (TFs) under heat stress

In shoots, G14 identified 652 and 827 differentially expressed TFs at 12 h and 24 h, respectively, while G9 identified 705 and 1,068 TFs. All TFs were classified into 40 families. The *ERF* family was the most abundant, followed by the *bHLH* family. Both up- and down-regulated TFs were mainly distributed in these two families (**Figure 7A**; **Supplementary Table 2**). The increased number of differentially expressed TFs at 24 h suggests that transcriptional regulation became more extensive during prolonged heat exposure.

**Figure 7:**
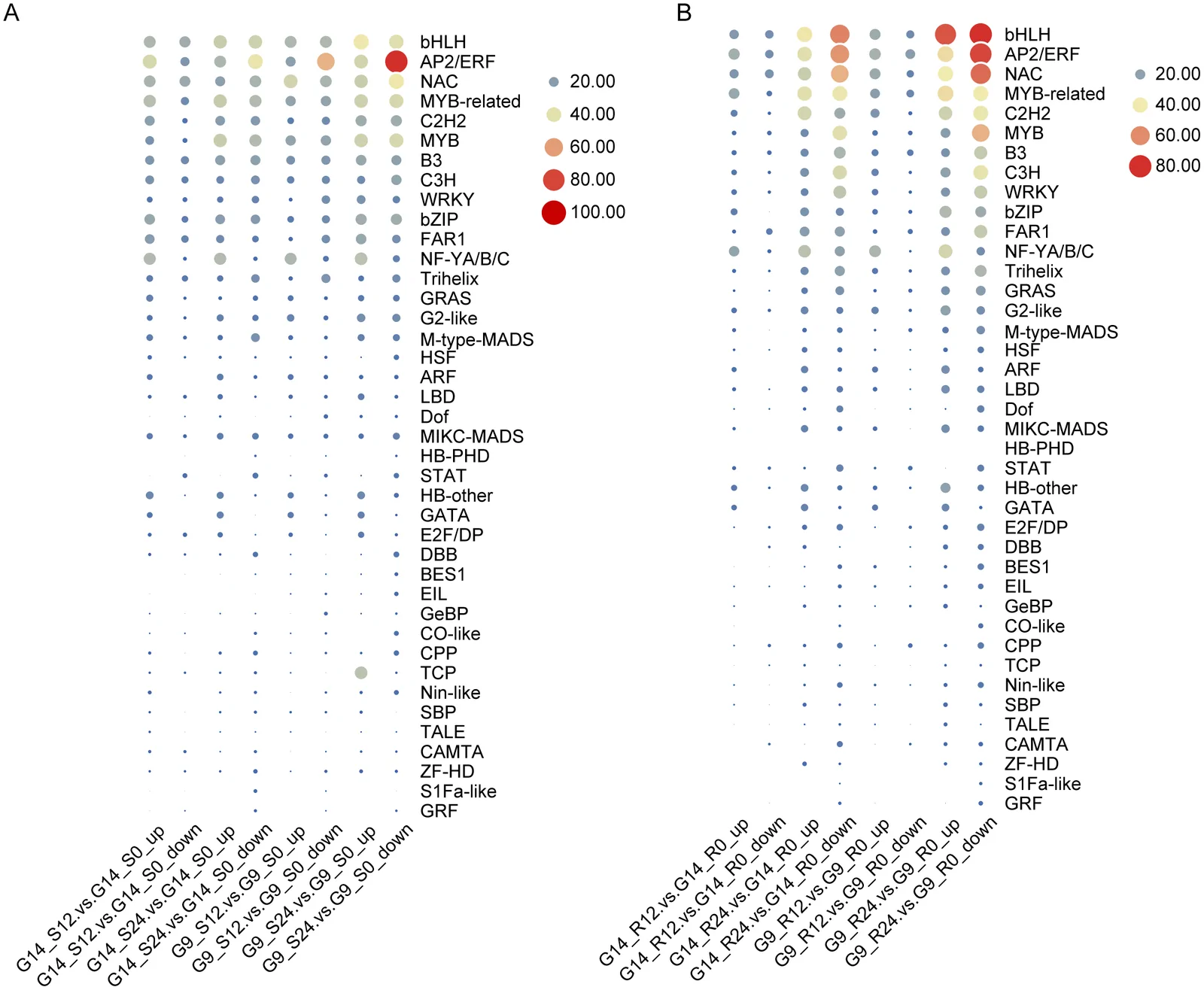
Differential transcription factor analysis in G14 and G9. **(A, B)** Heatmap showing differentially expressed TFs in shoots **(A)** and roots **(B)** of G14 and G9 at 12 h and 24 h of heat treatment.

In roots, G14 identified 377 and 1,068 TFs at 12 h and 24 h, respectively, whereas G9 identified 446 and 1,362 TFs. Similar to shoots, the *bHLH* and *ERF* families were dominant and accounted for a large proportion of both up- and down-regulated TFs (**Figure 7B**; **Supplementary Table 3**). The marked increase in differentially expressed TFs at 24 h indicates enhanced transcriptional responses at the later stage of heat stress.

In summary, the number of differentially expressed TFs increased over time in both shoots and roots, indicating dynamic changes in transcription factor regulation during heat stress. The consistent enrichment of *ERF* and *bHLH* family members suggests that these TF families may represent candidate transcription factors associated with the heat stress response in sesame. In addition, roots showed a greater number of differentially expressed TFs than shoots, indicating stronger transcriptional responses in roots under heat stress.

### 3.8 Validation of DEGs by RT−qPCR analysis

Integrated metabolomic and transcriptomic analyses revealed that glutathione metabolism was consistently associated with the heat stress response in sesame. Meanwhile, transcription factor analysis showed that members of the *ERF* and *bHLH* families exhibited differential expression patterns under heat stress. To validate the transcriptomic data, six genes were selected for RT-qPCR analysis, including two genes involved in glutathione metabolism (*105159842* and *105168386*) and four transcription factor genes belonging to the *ERF* and *bHLH* families (*ERF*: *105160373* and *105176892*; *bHLH*: *105165235* and *105161624*).

The expression patterns of the selected genes in the RNA-seq dataset are shown in the heatmap (**Figure 8A**). The two-glutathione metabolism-related genes and the selected *ERF* and *bHLH* transcription factor genes exhibited distinct expression responses under heat stress. RT-qPCR analysis showed that the expression trends of these genes were largely consistent with the RNA-seq results (**Figures 8B–G**). Although some differences in expression levels were observed between the two approaches, the overall expression patterns were comparable, supporting the reliability of the transcriptomic analysis. Together, these results indicate that genes associated with glutathione metabolism, as well as selected members of the *ERF* and *bHLH* transcription factor families, show differential expression in response to heat stress during sesame germination. These genes may serve as candidate genes for further investigation of the molecular mechanisms underlying heat tolerance in sesame.

**Figure 8:**
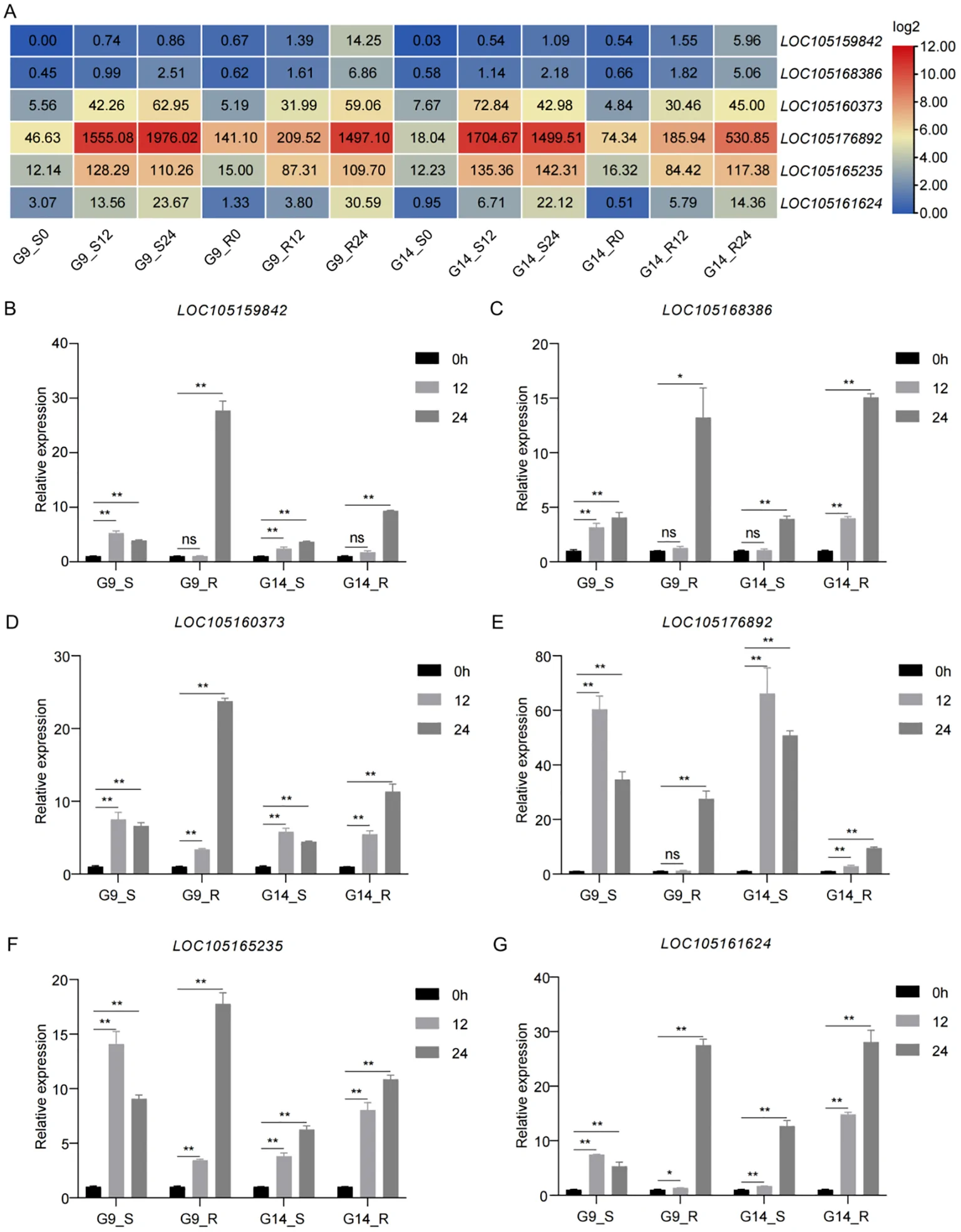
Expression validation of selected heat-responsive genes by RT-qPCR. **(A)** Heatmap showing the expression patterns of six selected genes based on RNA-seq data. The upper two genes are involved in glutathione metabolism, followed by *ERF* and *bHLH* transcription factor genes. **(B–G)** Validation of the selected genes by RT-qPCR. Significant differences were analyzed by Student’s *t*-test (mean ± SD; *p < 0.05; **p < 0.01; ns, not significant).

## 4 Discussion

Heat stress is a major factor that limits plant growth and yield (Verma et al., 2020; Aouz et al., 2023). It disrupts membrane stability, protein folding, and cellular redox balance (Wahid et al., 2007). As a result, photosynthesis is reduced, stomata close, and transpiration is altered (Yang et al., 2023). In this study, G14 showed better growth than G9 under both normal and high-temperature conditions. This difference became more obvious under heat stress (**Figure 1**). The maintenance of greater shoot and root growth in G14 under heat stress indicates that this genotype was better able to sustain growth-related physiological functions under high temperature. The lower H_2_O_2_ accumulation in G14 further suggests that reduced oxidative stress was associated with this difference in heat response.

Metabolomic analysis showed that heat stress caused large-scale metabolic changes in both genotypes (**Figures 2A–D**). However, the response patterns were clearly different. G14 showed fewer differential metabolites, whereas G9 showed a larger number of differential metabolites, indicating differences in the extent and pattern of metabolic reprogramming between the two genotypes (**Figures 2A–D**). Rather than reflecting a simple difference in stress severity, these patterns indicate that the two genotypes adjusted their metabolism in different ways under heat stress. Lipid metabolism was one of the key pathways enriched in G14. Lipids play an important role in maintaining membrane structure and fluidity under heat stress (Obata and Fernie, 2012; Liu et al., 2019; Dutta et al., 2025). The early and sustained changes in lipid-related metabolic pathways in G14 may be associated with its response to heat stress, although their direct contribution to membrane protection was not evaluated in this study. In contrast, G9 showed less consistent enrichment in lipid-related pathways, indicating a different pattern of lipid metabolic adjustment. Thus, the sustained lipid-related metabolic response in G14 may reflect a more coordinated adjustment of cellular metabolism during heat exposure, although whether these changes directly contribute to membrane stability remains to be determined.

Antioxidant metabolism was also important. Both genotypes showed enrichment in glutathione metabolism and related pathways (**Figure 3**; **Supplementary Figure 1**). However, G14 activated these pathways earlier and maintained them over time. Heat stress often leads to the accumulation of ROS, which can damage proteins, lipids, and DNA (Kan et al., 2023). Efficient removal of ROS is therefore critical for stress tolerance. The combination of sustained enrichment of antioxidant-related pathways and lower H_2_O_2_ accumulation in G14 provides physiological and metabolic evidence that redox regulation was an important component of its heat response. This coordinated antioxidant response may help G14 maintain cellular homeostasis during heat exposure. In addition, G14 showed consistent enrichment in secondary metabolism, including phenylpropanoid and flavonoid biosynthesis (**Figure 3**; **Supplementary Figure 1**). These compounds can act as antioxidants and help protect cells under stress (Blokhina et al., 2003; Nakabayashi et al., 2014; Dong and Lin, 2020; Jan et al., 2022). The enrichment of these pathways therefore suggests that secondary metabolism may complement the antioxidant response in G14 by increasing the metabolic capacity for stress-associated secondary compounds. However, the contribution of individual metabolites to heat tolerance was not directly tested. In contrast, G9 showed stronger enrichment in hormone signaling and energy metabolism pathways (**Figure 3**; **Supplementary Figure 1**). This pattern suggests that G9 underwent broader metabolic adjustments involving stress signaling and energy metabolism. These changes indicate that G9 responded to heat stress through substantial reorganization of signaling and energy-related metabolism, whereas G14 showed a more sustained response involving antioxidant, lipid-related, and secondary metabolic pathways.

Transcriptomic analysis also revealed distinct response patterns between the two genotypes. G9 showed more DEGs and larger changes than G14 (**Figure 4B**), whereas G14 showed fewer differentially expressed genes. The difference in DEG numbers was accompanied by differences in pathway enrichment, indicating distinct patterns of transcriptional reprogramming rather than a single uniform response to heat stress. KEGG analysis further showed that G14 showed sustained enrichment of key processes such as photosynthesis and protein processing (**Figure 5**). These processes are essential for plant survival under stress. Their continued regulation in G14 is consistent with the better maintenance of growth observed under heat stress and suggests that processes related to energy capture and protein homeostasis remained actively regulated in this genotype. In contrast, G9 mainly activated defense and signaling pathways (**Figure 5**), indicating broader transcriptional adjustments in response to heat stress. This broader response may reflect greater reorganization of cellular processes in G9, although the present data do not establish whether these transcriptional changes were beneficial or detrimental. Overall, the transcriptomic results indicate that G14 and G9 differed not simply in the magnitude of their responses, but in the cellular processes emphasized during heat exposure.

The integrated analysis of metabolome and transcriptome further showed that several biological processes were represented at both molecular levels under heat stress. In G14, key pathways, including lipid metabolism, antioxidant systems, and secondary metabolism, were consistently regulated at both the transcriptional and metabolic levels (**Figure 6**; **Supplementary Figure 2**). The recurrence of these pathways across the two omics layers suggests coordinated regulation of metabolism and gene expression, particularly for redox balance and secondary metabolism. Such coordination may contribute to a more consistent response to heat stress, although its effects on regulatory efficiency or energy cost were not directly measured. In contrast, G9 showed a different degree and pattern of correspondence between transcriptional and metabolic changes (**Figure 6**; **Supplementary Figure 2**), with greater representation of hormone signaling and energy-related pathways. These differences suggest that the two genotypes relied on different combinations of regulatory and metabolic processes during heat stress.

Transcription factors play central roles in plant stress responses (Baillo et al., 2019). In this study, the *ERF* and *bHLH* families were the most abundant and showed strong differential expression (**Figure 7**). The AP2/ERF family is a large group of plant-specific transcription factors that regulate growth, development, and stress adaptation (Wang et al., 2024). Previous studies in rice have shown that, overexpression of *OsERF115/AP2EREBP110* enhances heat tolerance during the vegetative stage by upregulating the proline biosynthesis gene *P5CS1* and increasing proline accumulation (Park et al., 2021). In addition, *ERF74/77* and *ERF108/125* improve heat tolerance through coordinated regulation of heat-responsive genes (Luo et al., 2025). Overexpression of *ERF95* or *ERF97* has also been shown to enhance basal heat tolerance in plants (Huang et al., 2021). Similarly, overexpression of *LlERF012* increases heat tolerance in both *Arabidopsis* and lily, whereas silencing this gene reduces heat tolerance (Li et al., 2024).

The *bHLH* family is also one of the largest groups of transcription factors in plants and is involved in development, secondary metabolism, and stress responses (Wu et al., 2024). *LlbHLH87* is rapidly induced by heat stress, and its overexpression enhances thermotolerance, while its silencing reduces heat tolerance (Wu et al., 2024). In rice, mutants of *bHLH110* show increased sensitivity to heat stress, whereas overexpression lines display improved tolerance (Lu et al., 2025). Together, these previous studies indicate that *ERF* and *bHLH* proteins can participate in heat-responsive processes in plants. In sesame, the strong differential expression of these families identifies them as candidate transcriptional components associated with the heat stress response. Their enrichment in the transcriptomic dataset, together with the contrasting physiological and metabolic responses of G14 and G9, makes these families relevant candidates for further investigation. However, because their functions were not directly tested in this study, their specific contributions to sesame heat tolerance remain to be determined.

Despite these findings, several limitations should be noted. First, only two genotypes were analyzed, which may not capture the full range of heat responses in sesame. In addition, the relatively limited number of biological samples and the experimental design restricted the application of network-based analyses such as weighted gene co-expression network analysis (WGCNA) in this study. Therefore, potential co-expression modules and their relationships with heat-responsive traits could not be systematically evaluated, and this should be considered a limitation of the present study. Second, although metabolomic and transcriptomic analyses identified key pathways, the functions of candidate genes and metabolites were not experimentally verified. In addition, this study focused on short-term heat stress, and the effects of prolonged high temperature were not assessed.

Future work should include more genotypes with different levels of heat tolerance to test whether these results are broadly applicable. Functional validation of candidate genes, especially those involved in lipid metabolism, antioxidant systems, and transcription factors such as *ERF* and *bHLH*, will be important to define their roles in heat tolerance. Moreover, integrating multi-omics data with physiological measurements, particularly indicators of membrane stability and oxidative damage, will provide a more complete understanding of heat stress responses and help determine whether the candidate metabolic and transcriptional processes identified here contribute directly to heat tolerance in sesame.

## 5 Conclusions

In summary, our results delineate thermotolerance strategies employed by sesame genotypes during germination. G14 confers heat resilience primarily through the early and sustained activation of lipid metabolism, antioxidant defenses, and secondary metabolic pathways, thereby maintaining homeostasis in core physiological processes such as photosynthesis and protein processing. In contrast, G9 exhibits a broader regulatory shift toward hormone signaling, carbon and energy metabolism, and vitamin biosynthesis, prioritizing defense activation over growth maintenance. Notably, the differential expression of *ERF* and *bHLH* transcription factor members suggests that they may be associated with genotype-specific responses to heat stress. Our findings provide insights into the molecular responses underlying the differences in heat tolerance between G14 and G9 and identify candidate genes and pathways for further investigation and the breeding of heat-tolerant sesame cultivars under climate change.

## Data Availability

The datasets presented in this study can be found in online repositories. The names of the repository/repositories and accession number(s) can be found below: https://ngdc.cncb.ac.cn/gsa, CRA041103.
