# Supplementary material for: Combined transcriptomics and metabolomics analysis reveals the molecular responses of heat tolerance during germination stage in sesame
Source: Front Plant Sci. 2026 Sep 9;17:1927626. doi: 10.3389/fpls.2026.1927626 (PMC13617605; doi:10.3389/fpls.2026.1927626)
Supplement: Supplementary file 1 [file DataSheet1.docx]

Supplementary Material

**Supplementary Figure 1.** KEGG enrichment analysis of differential metabolites in G14 and G9 in negative ion mode. A-B. KEGG enrichment bubble plots in shoots after 12 h (A) and 24 h (B) of heat treatment. C-D. KEGG enrichment bubble plots in roots after 12 h (C) and 24 h (D).

**Supplementary Figure 2.** Integrated KEGG enrichment analysis of metabolome and transcriptome in negative ion mode. A-B. Integrated KEGG enrichment bubble plots in shoots after 12 h (A) and 24 h (B). C-D. Integrated KEGG enrichment bubble plots in roots after 12 h (C) and 24 h (D).

**Supplementary Figure 3.** KEGG enrichment analysis of differential metabolites in G14 and G9 in positive ion mode. A-B. KEGG enrichment bubble plots of differential metabolites in roots of G14 and G9 after 12 h (A) and 24 h (B) of heat treatment.

**Supplementary Figure 4.** KEGG enrichment analysis of DEGs in G14 and G9. A-B. KEGG enrichment bubble plots of DEGs in roots of G14 and G9 after 12 h (A) and 24 h (B) of heat treatment.

**Supplementary Figure 5.** Integrated KEGG enrichment analysis of metabolome and transcriptome in positive ion mode. A-B. Integrated KEGG enrichment bubble plots in roots of G14 and G9 after 12 h (A) and 24 h (B).

**Supplementary Table 1.** Transcriptome sequencing quality

| **sample** | **library** | **raw_reads** | **raw_bases** | **clean_reads** | **clean_bases** | **Alignment Rate** | **Q20 (%)** | **Q30 (%)** | **GC_pct** |
| --- | --- | --- | --- | --- | --- | --- | --- | --- | --- |
| G14_R0_1 | FRAS250047118-3r | 45346036 | 6.8G | 44075824 | 6.61G | 89.27% | 99.1 | 97.21 | 45.47 |
| G14_R0_2 | FRAS250047118-2r | 43738626 | 6.56G | 42754238 | 6.41G | 89.13% | 99.05 | 97.08 | 45.39 |
| G14_R0_3 | FRAS250047118-1r | 44346370 | 6.65G | 43448882 | 6.52G | 89.61% | 99.07 | 97.1 | 45.31 |
| G14_R12_1 | FRAS250047119-3r | 47768814 | 7.17G | 45981066 | 6.9G | 90.85% | 99.1 | 97.24 | 45.21 |
| G14_R12_2 | FRAS250047119-2r | 44356562 | 6.65G | 43250562 | 6.49G | 91.63% | 99.1 | 97.19 | 45.29 |
| G14_R12_3 | FRAS250047119-1r | 42442950 | 6.37G | 41374896 | 6.21G | 91.71% | 99.11 | 97.25 | 45.33 |
| G14_R24_1 | FRAS250047120-1r | 45847208 | 6.88G | 43678398 | 6.55G | 89.3% | 99.07 | 97.11 | 45 |
| G14_R24_2 | FRAS250047120-2r | 42622714 | 6.39G | 38663814 | 5.8G | 90.94% | 99.07 | 97.11 | 44.97 |
| G14_R24_3 | FRAS250047120-3r | 45081022 | 6.76G | 42903684 | 6.44G | 88.45% | 99.1 | 97.24 | 45.2 |
| G14_S0_1 | FRAS250047124-1r | 45916418 | 6.89G | 43987186 | 6.6G | 90.94% | 99.07 | 97.12 | 45.47 |
| G14_S0_2 | FRAS250047124-2r | 45278822 | 6.79G | 41444738 | 6.22G | 93.34% | 99.08 | 97.13 | 45.65 |
| G14_S0_3 | FRAS250047124-3r | 48654322 | 7.3G | 46013902 | 6.9G | 90.04% | 99.11 | 97.26 | 45.42 |
| G14_S12_1 | FRAS250047125-1r | 40628674 | 6.09G | 38122136 | 5.72G | 90.0% | 99.06 | 97.11 | 45.84 |
| G14_S12_2 | FRAS250047125-2r | 47530752 | 7.13G | 44517552 | 6.68G | 90.49% | 99.07 | 97.09 | 45.78 |
| G14_S12_3 | FRAS250047125-3r | 41165142 | 6.17G | 38429102 | 5.76G | 91.39% | 99.05 | 97.11 | 45.97 |
| G14_S24_1 | FRAS250047126-2r | 43403974 | 6.51G | 40498110 | 6.07G | 91.2% | 99.06 | 97.12 | 45.7 |
| G14_S24_2 | FRAS250047126-1r | 47729002 | 7.16G | 44052170 | 6.61G | 90.27% | 99.1 | 97.22 | 45.36 |
| G14_S24_3 | FRAS250047126-3r | 47954822 | 7.19G | 44351824 | 6.65G | 90.4% | 99.1 | 97.23 | 45.46 |
| G9_R0_1 | FRAS250047121-2r | 41575880 | 6.24G | 40525740 | 6.08G | 85.76% | 99.12 | 97.27 | 44.97 |
| G9_R0_2 | FRAS250047121-3r | 49702358 | 7.46G | 48124360 | 7.22G | 88.29% | 99.1 | 97.24 | 45.23 |
| G9_R0_3 | FRAS250047121-1r | 44666504 | 6.7G | 43319942 | 6.5G | 88.61% | 99.04 | 97.02 | 45.37 |
| G9_R12_1 | FRAS250047122-3r | 52666922 | 7.9G | 50248256 | 7.54G | 90.28% | 98.87 | 96.57 | 45.16 |
| G9_R12_2 | FRAS250047122-2r | 43820000 | 6.57G | 42324806 | 6.35G | 90.26% | 99.07 | 97.1 | 45.31 |
| G9_R12_3 | FRAS250047122-1r | 44813922 | 6.72G | 43167872 | 6.48G | 90.3% | 99.11 | 97.23 | 45.46 |
| G9_R24_1 | FRAS250047123-3r | 48583656 | 7.29G | 46428564 | 6.96G | 90.71% | 99.15 | 97.33 | 44.63 |
| G9_R24_2 | FRAS250047123-1r | 43028048 | 6.45G | 41098090 | 6.16G | 90.85% | 99.11 | 97.25 | 44.98 |
| G9_R24_3 | FRAS250047123-2r | 48298516 | 7.24G | 46172638 | 6.93G | 90.84% | 99.12 | 97.25 | 44.99 |
| G9_S0_1 | FRAS250047127-2r | 48018090 | 7.2G | 43876108 | 6.58G | 86.6% | 99.07 | 97.17 | 45.3 |
| G9_S0_2 | FRAS250047127-3r | 52002792 | 7.8G | 47552894 | 7.13G | 90.88% | 99.04 | 97.1 | 45.47 |
| G9_S0_3 | FRAS250047127-1r | 47588918 | 7.14G | 43637580 | 6.55G | 91.85% | 99.08 | 97.15 | 45.56 |
| G9_S12_1 | FRAS250047128-2r | 48680712 | 7.3G | 45453122 | 6.82G | 91.85% | 99.11 | 97.24 | 45.35 |
| G9_S12_2 | FRAS250047128-1r | 48569618 | 7.29G | 44361706 | 6.65G | 90.83% | 99.12 | 97.28 | 45.47 |
| G9_S12_3 | FRAS250047128-3r | 48097740 | 7.21G | 45030138 | 6.75G | 92.54% | 99.14 | 97.3 | 46 |
| G9_S24_1 | FRAS250047129-2r | 44688054 | 6.7G | 41882474 | 6.28G | 91.46% | 99.17 | 97.38 | 45.53 |
| G9_S24_2 | FRAS250047129-1r | 46888788 | 7.03G | 43981426 | 6.6G | 91.08% | 99.11 | 97.22 | 45.45 |
| G9_S24_3 | FRAS250047129-3r | 45093038 | 6.76G | 42341714 | 6.35G | 92.53% | 99.09 | 97.2 | 45.44 |

**Supplementary Table 2.** Differentially expressed TFs in shoots

| **Transcription factor** | **S12vsS0 (G14)** | | **S24vsS0 (G14)** | | **S12vsS0 (G9)** | | **S24vsS0 (G9)** | |
| --- | --- | --- | --- | --- | --- | --- | --- | --- |
|  | **down** | **up** | **down** | **up** | **down** | **up** | **down** | **up** |
| bHLH | 25 | 28 | 35 | 34 | 28 | 28 | 39 | 43 |
| AP2/ERF | 18 | 37 | 41 | 28 | 55 | 21 | 85 | 36 |
| NAC | 24 | 27 | 27 | 21 | 27 | 36 | 43 | 34 |
| MYB-related | 15 | 30 | 29 | 34 | 22 | 22 | 37 | 35 |
| C2H2 | 8 | 21 | 19 | 20 | 15 | 11 | 25 | 25 |
| MYB | 7 | 17 | 29 | 34 | 22 | 22 | 37 | 35 |
| B3 | 15 | 18 | 21 | 21 | 18 | 17 | 21 | 23 |
| C3H | 12 | 17 | 17 | 14 | 15 | 13 | 22 | 15 |
| WRKY | 7 | 9 | 13 | 10 | 16 | 5 | 13 | 17 |
| bZIP | 11 | 23 | 19 | 20 | 15 | 11 | 25 | 25 |
| FAR1 | 13 | 20 | 11 | 15 | 16 | 6 | 16 | 23 |
| NF-YA/B/C | 5 | 29 | 6 | 29 | 9 | 28 | 12 | 31 |
| Trihelix | 10 | 11 | 16 | 10 | 18 | 6 | 15 | 9 |
| GRAS | 4 | 12 | 8 | 5 | 5 | 9 | 11 | 10 |
| G2-like | 4 | 9 | 10 | 12 | 7 | 13 | 15 | 16 |
| M-type-MADS | 5 | 11 | 17 | 8 | 5 | 7 | 12 | 12 |
| HSF | 3 | 6 | 3 | 4 | 4 | 3 | 8 | 2 |
| ARF | 0 | 9 | 5 | 11 | 6 | 9 | 6 | 6 |
| LBD | 5 | 5 | 3 | 8 | 5 | 6 | 5 | 11 |
| Dof | 2 | 1 | 1 | 3 | 6 | 2 | 4 | 4 |
| MIKC-MADS | 5 | 10 | 11 | 11 | 7 | 8 | 12 | 8 |
| HB-PHD | 1 | 0 | 3 | 0 | 2 | 1 | 3 | 0 |
| STAT | 7 | 1 | 9 | 1 | 5 | 2 | 8 | 2 |
| HB-other | 2 | 14 | 3 | 12 | 3 | 10 | 7 | 14 |
| GATA | 0 | 9 | 1 | 12 | 3 | 10 | 3 | 12 |
| E2F/DP | 6 | 5 | 2 | 8 | 2 | 6 | 4 | 10 |
| DBB | 3 | 4 | 8 | 3 | 1 | 2 | 9 | 2 |
| BES1 | 1 | 0 | 2 | 1 | 2 | 2 | 5 | 2 |
| EIL | 0 | 1 | 1 | 0 | 3 | 2 | 6 | 2 |
| GeBP | 1 | 2 | 2 | 2 | 5 | 1 | 3 | 2 |
| CO-like | 2 | 2 | 4 | 0 | 3 | 2 | 7 | 0 |
| CPP | 1 | 4 | 7 | 4 | 3 | 2 | 8 | 3 |
| TCP | 3 | 3 | 4 | 3 | 1 | 2 | 3 | 31 |
| Nin-like | 0 | 5 | 4 | 3 | 3 | 1 | 7 | 4 |
| SBP | 1 | 3 | 4 | 3 | 3 | 3 | 1 | 4 |
| TALE | 1 | 3 | 2 | 2 | 1 | 2 | 2 | 2 |
| CAMTA | 4 | 4 | 4 | 2 | 2 | 1 | 3 | 3 |
| ZF-HD | 3 | 3 | 6 | 3 | 4 | 2 | 4 | 5 |
| S1Fa-like | 1 | 1 | 5 | 0 | 2 | 0 | 1 | 0 |
| GRF | 2 | 1 | 4 | 0 | 2 | 0 | 3 | 0 |

**Supplementary Table 3.** Differentially expressed TFs in roots

| **Transcription factor** | **R12vsR0(G14)** | | **R24vsR0(G14)** | | **R12vsR0(G9)** | | **R24vsR0(G9)** | |
| --- | --- | --- | --- | --- | --- | --- | --- | --- |
|  | **down** | **up** | **down** | **up** | **down** | **up** | **down** | **up** |
| bHLH | 15 | 18 | 62 | 41 | 14 | 23 | 80 | 72 |
| AP2/ERF | 15 | 24 | 58 | 37 | 19 | 25 | 75 | 43 |
| NAC | 16 | 17 | 52 | 32 | 12 | 23 | 67 | 40 |
| MYB-related | 8 | 22 | 39 | 37 | 13 | 19 | 40 | 43 |
| C2H2 | 4 | 11 | 23 | 34 | 5 | 17 | 39 | 33 |
| MYB | 6 | 6 | 37 | 14 | 6 | 8 | 52 | 19 |
| B3 | 6 | 7 | 26 | 14 | 10 | 11 | 31 | 16 |
| C3H | 4 | 8 | 35 | 16 | 4 | 12 | 38 | 20 |
| WRKY | 4 | 8 | 30 | 13 | 4 | 10 | 31 | 17 |
| bZIP | 1 | 11 | 14 | 16 | 5 | 8 | 23 | 27 |
| FAR1 | 10 | 5 | 20 | 19 | 7 | 6 | 31 | 16 |
| NF-YA/B/C | 5 | 21 | 20 | 29 | 3 | 26 | 15 | 34 |
| Trihelix | 3 | 5 | 20 | 14 | 4 | 9 | 26 | 16 |
| GRAS | 2 | 3 | 16 | 10 | 5 | 4 | 19 | 16 |
| G2-like | 4 | 8 | 11 | 13 | 4 | 12 | 15 | 20 |
| M-type-MADS | 1 | 5 | 8 | 6 | 5 | 3 | 15 | 10 |
| HSF | 2 | 3 | 7 | 8 | 2 | 3 | 10 | 6 |
| ARF | 0 | 7 | 6 | 12 | 2 | 9 | 8 | 15 |
| LBD | 2 | 5 | 10 | 9 | 2 | 6 | 12 | 15 |
| Dof | 2 | 2 | 11 | 4 | 2 | 1 | 12 | 2 |
| MIKC-MADS | 0 | 4 | 7 | 12 | 1 | 6 | 10 | 15 |
| HB-PHD | 0 | 0 | 0 | 0 | 0 | 0 | 0 | 0 |
| STAT | 4 | 5 | 12 | 3 | 6 | 3 | 11 | 1 |
| HB-other | 3 | 9 | 7 | 13 | 3 | 6 | 10 | 20 |
| GATA | 0 | 8 | 4 | 12 | 0 | 9 | 4 | 13 |
| E2F/DP | 3 | 2 | 10 | 7 | 4 | 2 | 12 | 7 |
| DBB | 3 | 0 | 2 | 5 | 2 | 0 | 9 | 7 |
| BES1 | 1 | 1 | 6 | 2 | 2 | 4 | 10 | 4 |
| EIL | 2 | 2 | 5 | 2 | 2 | 3 | 8 | 5 |
| GeBP | 0 | 2 | 3 | 4 | 3 | 2 | 3 | 6 |
| CO-like | 0 | 1 | 3 | 0 | 0 | 0 | 7 | 0 |
| CPP | 4 | 2 | 8 | 4 | 6 | 2 | 10 | 4 |
| TCP | 2 | 1 | 2 | 3 | 1 | 1 | 3 | 3 |
| Nin-like | 1 | 2 | 8 | 3 | 2 | 3 | 9 | 5 |
| SBP | 1 | 2 | 3 | 5 | 0 | 2 | 4 | 6 |
| TALE | 1 | 1 | 3 | 2 | 1 | 2 | 2 | 5 |
| CAMTA | 3 | 0 | 9 | 1 | 3 | 1 | 6 | 5 |
| ZF-HD | 0 | 0 | 3 | 6 | 0 | 0 | 4 | 4 |
| S1Fa-like | 0 | 0 | 2 | 0 | 0 | 0 | 4 | 0 |
| GRF | 1 | 0 | 4 | 0 | 0 | 1 | 6 | 1 |

**Supplementary Table 4.** Primer sequences for the DEGs used in qRT − PCR assays and relative changes in expression.

| **Gene ID** | **Forward Sequence** | **Reverse Sequence** |
| --- | --- | --- |
| LOC105159842 | CACTGCGGTTGGAATTCGTG | ACGAGCCCAATCCTTCACAG |
| LOC105168386 | GCCGTGATCCCGTATCCTGATG | GAGCCACCTGTTCTGCCACAA |
| LOC105160373 | CTGCTCAGGAGCTTGTGGAGAG | CCATGCGTAATGGACCGAACCT |
| LOC105176892 | CGAGGTGGTTCTGCTGGATGTA | GAGCGACTCACAGACGGGTT |
| LOC105165235 | CGATGACGCTGTTGTGGAAGAG | ACCACCCGCTCACATTAGGC |
| LOC105161624 | CAATCCCATCGCCGACTCTTTG | CCTCTCCGCTGCTCCAACAT |
| SiUBQ6 | CACCAAGCCGAAGAAGATCAAG | CCTCAGCCTCTGCACCTTTC |
